# A Network Access Control Framework for 6LoWPAN Networks

**DOI:** 10.3390/s130101210

**Published:** 2013-01-18

**Authors:** Luís M. L. Oliveira, Joel J. P. C. Rodrigues, Amaro F. de Sousa, Jaime Lloret

**Affiliations:** 1 Instituto de Telecomunicações, Universidade da Beira Interior, Rua Marquês d'Ávila e Bolama, 6201-001 Covilhã, Portugal; E-Mail: loliveira@ipt.pt; 2 Instituto Politécnico de Tomar, Quinta do Contador, Estrada da Serra, 2300-313 Tomar, Portugal; 3 Instituto de Telecomunicações, Universidade de Aveiro, Campus Universitário de Santiago, 3810-193 Aveiro, Portugal; E-Mail: asou@ua.pt; 4 Instituto de Investigación para la Gestión Integrada de Zonas Costeras, Universidad Politécnica de Valencia, C/Paranimf, n° 1, 46730 Grao de Gandia, Spain; E-Mail: jlloret@dcom.upv.es

**Keywords:** wireless sensor networks, WSN, 6LoWPAN, network access control

## Abstract

Low power over wireless personal area networks (LoWPAN), in particular wireless sensor networks, represent an emerging technology with high potential to be employed in critical situations like security surveillance, battlefields, smart-grids, and in e-health applications. The support of security services in LoWPAN is considered a challenge. First, this type of networks is usually deployed in unattended environments, making them vulnerable to security attacks. Second, the constraints inherent to LoWPAN, such as scarce resources and limited battery capacity, impose a careful planning on how and where the security services should be deployed. Besides protecting the network from some well-known threats, it is important that security mechanisms be able to withstand attacks that have not been identified before. One way of reaching this goal is to control, at the network access level, which nodes can be attached to the network and to enforce their security compliance. This paper presents a network access security framework that can be used to control the nodes that have access to the network, based on administrative approval, and to enforce security compliance to the authorized nodes.

## 1. Introduction

Low power wireless personal area networks (LoWPAN) [1] comprise devices compliant with the IEEE 802.15.4 [2] standard and are widely used in embedded applications such as environmental monitoring, smart-grids, surveillance, industrial and home automation. These applications often require a large number of small devices to cover large areas and must operate unattended for years equipped with small batteries. Many of the IEEE 802.15.4 compliant devices are characterized by small size, power constraints, small computing and storage resources and by reduced radio ranges and throughput. Self-organization, fault-tolerance, and self-optimization are the main characteristics of LoWPAN networks [2].

Initially, the IP protocol stack was considered too complex to be supported by IEEE 802.15.4 devices. Meanwhile, the scientific community, together with the industry, started to rethink many of the misconceptions about the use of IP protocol stacks in resource-constrained devices [3]. Now, the IPv6 protocol is the most consensual solution to connect LoWPAN networks to the Internet facilitating the design and deployment of applications [4]. However, IPv6 was not designed to be used in such devices and, therefore, an adaptation layer was introduced between the link layer and the network layer. This adaptation layer, proposed by IETF 6LoWPAN Working Group [1], enables the transmission of IPv6 datagrams over IEEE 802.15.4 links by providing header compression (to reduce overhead), fragmentation (to support the IPv6 minimum MTU requirement) and support for layer-two forwarding (to deliver IPv6 datagram over multiple radio hops). Although the standard IPv6 neighbor discovery protocol [5] might work on 6LoWPAN networks, it exhibits high overhead and includes no security support. The lightweight secure neighbor discovery optimizations for 6LoWPAN protocol (the LSEND protocol) [6,7] was proposed to circumvent these problems.

LoWPAN networks exhibit a large number of vulnerabilities, which make them even more prone to security attacks [8–12] than traditional IP networks. In fact, a single LoWPAN network can scale up to thousands of nodes without any fixed infrastructure and these nodes are often installed in harsh and unattended environments. Moreover, the addition of new nodes makes the network topology dynamic and complex to manage. Several security mechanisms have been proposed, some of them defined to address some particular well-known attacks [13]. Besides protecting the network from well-known attacks, it is also important that security mechanisms be able to withstand attacks that have not been identified before [14]. If a malicious node is prevented from becoming attached to the network, it cannot communicate with any network element and, therefore, it cannot launch any type of security attack. Therefore, one way of reaching this goal is to control, at the network access level, which nodes can be attached to the network and to enforce their security compliance. Besides the security advantages, such methodology also makes the network more manageable, while increasing its reliability and extending its lifetime [15].

This paper proposes a network access control security framework for 6LoWPAN networks, that controls the access of nodes to the network, based on administrative authorization, and enforces security compliance to the authorized nodes. The proposed framework makes use of LSEND protocol (for secure neighbor discovery and key pairwise generation), RPL (for datagram routing), and Seluge (for security compliant code dissemination). Unlike other access control mechanisms, this proposal includes an automatic remediation mechanism to enable nodes to become security compliant, if necessary, in order to have their access granted by the network.

The remainder of this paper is organized as follows: Section 2 addresses the security support requirements for LoWPAN networks and reviews the current solutions. Section 3 focuses on the technologies and protocols used to support the proposed network access control security framework. The Sections 4 and 5 present the proposed network access control design framework and discuss its application and the requirements for its implementation. Finally, Section 6 concludes the paper and identifies future research topics.

## 2. Security on LoWPAN Networks

Note that both LoWPAN networks and general resource unconstrained networks share almost the same security requirements [12,16]. However, due to the node resource constraints, the number of nodes and the absence of an organized communication infrastructure, supporting security services in LoWPAN networks is more challenging when compared with resource unconstrained networks. Confidentiality, authenticity, integrity, availability, data freshness, robustness, and survivability are the most relevant security requirements in LoWPAN networks [17,18]. Confidentiality ensures that only legitimate entities have access to the data transmitted and stored in the network nodes. Authenticity ensures that data is actually provided by its source nodes. Integrity guarantees that no data is changed by any other entity, without being detected. Availability ensures that services provision is always available to their legitimate users. Data freshness prevents other entities from replaying old messages. Network robustness and survivability guarantees that the network still works properly even in the presence of intrusions, attacks, accidents and failures.

Following [19], LoWPAN security attacks can be broadly classified as external *versus* internal attacks, passive *versus* active attacks and mote-class *versus* laptop-class attacks. In external attacks, the attacker device can only use its own resources to perform the attack and has no access to the resources of the other LoWPAN nodes. External attacks can be prevented with cryptography. For example, a cryptographic mechanism used to support authentication and confidentiality prevents an external attacker from eavesdropping third-party messages or injecting false messages. In internal attacks, the attacker device is able to compromise legitimate nodes by using their resources to perform the attacks. Internal attacks involve either the injection of malicious code into target nodes (exploiting flaws in the application modules) and/or the access of key material code and data of the target nodes. This type of attack enables the attacker to appear as a legitimate node in the network gaining the trust of the other legitimate nodes. Internal attacks are much harder to detect when compared with external attacks [20].

Passive attacks are based on information gathering without modification. Eavesdropping and monitoring are examples of passive attacks. The active attacks involve modifications on the legitimate data stream or false data injection. In the mote-class attacks, nodes with similar resources as the legitimate nodes are used to perform the attack. In laptop-class attacks, an attacker uses devices with more resources, such as laptops, to perform the attack. Laptop-class attacks are especially effective, for example, to jam the wireless channel.

Security attacks against LoWPAN networks can also be classified, according to security requirements, in the following three main groups [16]: attacks against secrecy and authenticity, attacks against network availability, and stealthy attacks (*i.e*., attacks against service integrity). Eavesdropping, packet replay, tampering and spoofing are examples of attacks against the secrecy and authenticity. Denial of service (DoS) is the main example of an attack against the network availability and can be targeted at the different layers of the networking stack [9,10]. In the stealthy attacks, the main goal is to make a legitimate node to accept false data values generated by the attacker and, in this way, the compromised legitimate node can also be used to amplify the dissemination of false data through all other legitimate nodes. Several mechanisms, most of them based on cryptography, can be used to address these different requirements in LoWPAN networks. Currently, the research on providing security solutions for LoWPAN networks has been focused mainly in three categories: (i) key management, (ii) authentication and secure routing and (iii) secure services. Some solutions were proposed to establish and manage cryptographic keys between nodes to enable authentication and encryption mechanisms [21] while others have been proposed to protect routing protocols [22]. Progress has been achieved on specialized secure services, such as secure localization, secure data aggregation and secure time synchronization [23,24].

There are also many proposals of security mechanisms addressing only particular attacks or used in particular layers as security tools. However, the major disadvantage with single layer security approaches is that security mechanisms are introduced on each layer, which in most of the cases tends to overall solutions with waste of resources power and exaggerated delays on message forwarding. Recently, researchers are pursuing security-integrated systems instead of concentrating on particular attacks or layered based mechanisms. Some frameworks have been proposed to address simultaneously more than one security attack or to mitigate attacks using more than one security mechanism [25]. The most significant proposal is Security Protocols for Sensor Networks (SPINS) [26] which is composed by the Secure Network Encryption Protocol (SNEP) and micro-TESLA. SNEP provides data confidentiality and two-way data authentication with reduced overhead while micro-TESLA is a lightweight version of Time Efficient Streamed Loss-tolerant Authentication (TESLA) providing authenticated streaming broadcast.

SPINS does not cover, though, some relevant security issues, such as compromised nodes detection, DoS attacks or network and traffic analysis issues. Moreover, SPINS assumes a static network topology ignoring the ad hoc and mobile nature of LoWPAN networks. In [27], the authors propose a framework to provide secure cluster formation, security key management scheme and secure routing. It includes three components: a security mechanism to provide secrecy for communications in LoWPAN, an efficient session key distribution mechanism and a centralized key revocation scheme. The proposed framework does not depend on a specific key mechanism scheme and can be used to support many security applications, such as secure group communications.

Currently, the control of the access to the network is considered a critical security service in LoWPAN networks because it can be used to prevent malicious nodes from joining the network and launching internal attacks [15]. With such service, only eligible nodes can access the network, while queries from external attacker nodes are not answered or forwarded by regular nodes. In defining the recently proposed access control schemes, three main aspects can be distinguished: new node addition, authenticated querying and user authentication [14]. Query authentication schemes guarantee data origin authentication and data integrity while user authentication schemes are the basic solution used for the access control issue. New node addition schemes use mechanisms based on Elliptic Curve Cryptography (ECC) [28] to prevent malicious nodes from joining the network. Most of the secure network access systems provide node authentication and packet authentication, integrity verification and confidentiality. In [29], a self-certified elliptic curve Diffie-Hellman (ECDH) cryptosystem is used to establish a pairwise key between a new sensor node and a required Certificate Authority (CA) element, which can be implemented on regular nodes or on more powerful nodes such the border routers. The CA launches a two-way authentication procedure with the new node and establishes a pairwise key using the self-certified ECDH based protocol. The ECDH is used to guarantee nodes identification and to deliver a shared key to the new node. The shared key is the same for all nodes and is used to guarantee packet privacy and integrity. Note that the security of this scheme depends strongly on the secrecy of the shared key, *i.e.*, it fails if a single node is compromised.

To the best of our knowledge, though, none of the existing security frameworks includes the following important aspects which are dealt with by this proposal: to enable the node security compliance evaluation and enforcement (mitigating the security threats of internal attacks) and to use the same key pairwise for node authentication and routing protocol security.

## 3. Related Technologies

### 3.1. 6LoWPAN

Supporting IPv6 on sensor nodes simplifies simultaneously the task of connecting LoWPAN devices to the Internet and the application developing process. Currently, the IEEE 802.15.4 protocol is widely accepted as the PHY and MAC layer protocol for LoWPAN networks. Nevertheless, the network layer protocol must comply with the constraints imposed by the IEEE 802.15.4 protocol and the properties of the standard IPv6 protocol do not fully match with such constraints. Low bandwidth, low-power resources and the maximum packet size of 127 bytes are the most relevant characteristics of the IEEE 802.15.4 standard, which must be dealt with in proper protocol adaptation. Moreover, the support of standard IPv6 headers over LoWPAN would result in extremely small payloads for higher layer protocols. Besides LoWPAN requirements, it is also necessary to guarantee that 6LoWPAN is compliant with the IPv6 minimum MTU of 1280 bytes and, consequently, fragmentation and reassembly is required.

The IETF created the 6LoWPAN working group to define how to support IPv6 over IEEE 802.15.4 protocol. The 6LoWPAN working group was focused on the following issues [1]: (i) to define a neighbor discovery protocol fitted for low-power networks, (ii) to describe mechanisms allowing compression of 6LoWPAN headers in order to reduce the header overhead and (iii) to define the 6LoWPAN routing requirements and approaches. Two RFCs were released, the RFC 4919 [1] and the RFC 4944 [30]. The first document describes the assumptions, problem statement, and goals of 6LoWPAN. The second document proposes a 6LoWPAN adaptation layer (between IPv6 and IEEE 802.15.4) describing the frame format for transmission of IPv6 packets, the method for defining IPv6 link-local addresses and stateless auto configured addresses, the header compression and the frame delivery process in IEEE 802.15.4 mesh networks.

Rather than defining a single header (like IPv4), the 6LoWPAN uses stacked headers as the original IPv6 protocol does. The 6LoWPAN standard defines four header types: the dispatch header, the IPv6 compression header, the fragmentation header and the mesh header (by default, only the dispatch and compression headers are used). At the beginning of each header, a header type field identifies the header format.

**Figure 1.**
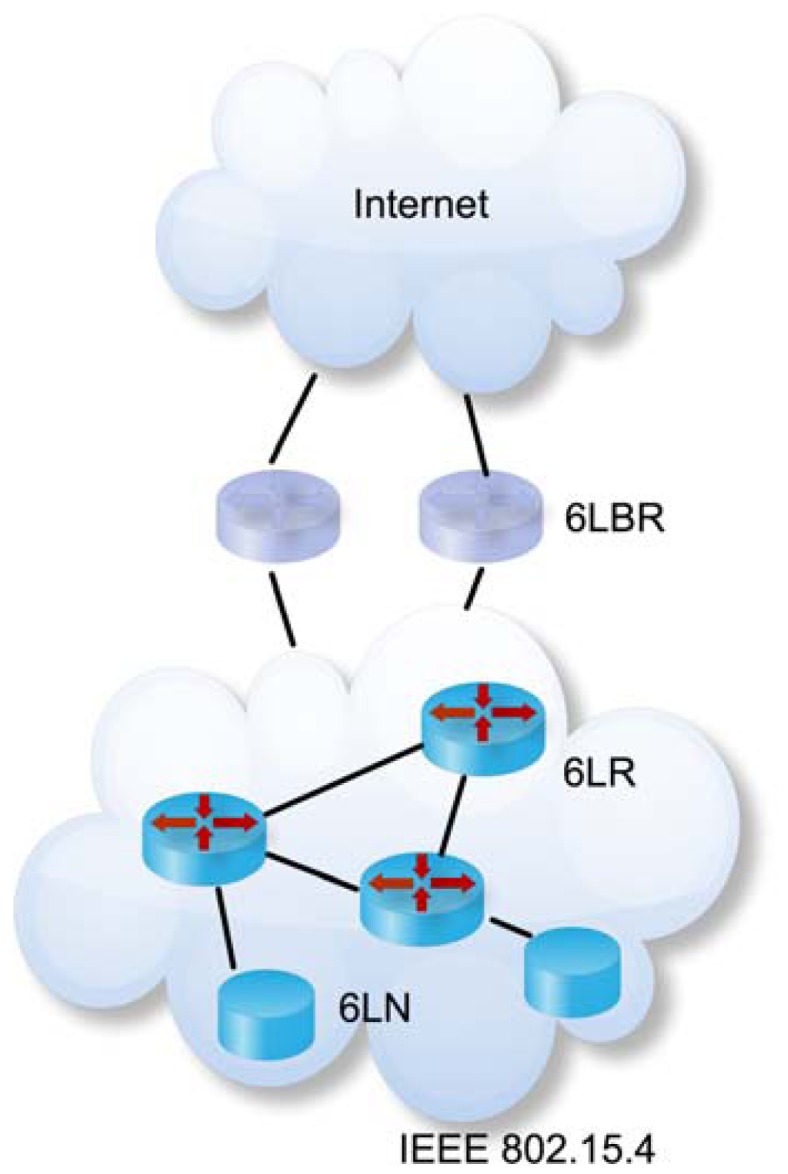
6LoWPAN network architecture.

An illustrated in Figure 1, a typical LoWPAN [31] consists of nodes (named 6LN or 6LoWPAN Nodes), routers (named 6LR or 6LoWPAN Routers) and border routers (named 6LBR or 6LoWPAN Border Routers).

Nodes (or 6LNs) usually do sensing and actuation operations but they do not forward datagrams form other nodes to their destination nodes. Routers (or 6LRs) are intermediate nodes that forward datagram from others nodes (or routers) to their destination nodes in the same LoWPAN and are present only in route-over topologies. Border routers (or 6LBRs) are the interconnection devices between the LoWPAN network and others networks as, for example, the Internet. Typically, nodes and routers have energy and computational resource constraints while border routers are mainly powered with much more computational resources.

### 3.2. Lightweight Secure Neighbor Discovery for 6LoWPAN (LSEND)

In traditional IPv6 networks, both nodes and routers use the neighbor discovery protocol [5] and the stateless address autoconfiguration [32], which, together, are referred to as the neighbor discovery protocols (NDP). These protocols enable the following functions: (i) learning prefixes and configuration parameters related to address configuration, (ii) locating neighborhood routers, (iii) maintaining reachability information on active neighbors and (iv) detecting duplicate addresses. Note that NDP was proposed for unconstrained node devices. Moreover, NDP for IPv6 networks uses multicast to exchange most of the protocol messages and was designed considering that routers and nodes are always active. Given the resource constraints of sensor nodes, the resource inefficiency associated to multicast based mechanisms, the low duty-cycle and multi-hop support, NDP on 6LoWPAN networks requires a different approach. Neighbor discovery optimization for low power and lossy networks [6] is a work in progress at IETF 6LoWPAN working group. It proposes optimizations to NDP, header compression context information dissemination, auto configuration addressing mechanisms and duplicate address detection for low power networks. The NDP signaling was changed by replacing the standard address resolution mechanism (based on multicast messages between hosts) with an address registration mechanism. Moreover, some multicast messages associated to node address configuration were replaced by unicast messages, providing host-initiated request for router advertisements (RA) and eliminating in this way the need for periodic router advertisement multicasting. In this way, NDP for 6LoWPAN is more suitable for multi-hop sensor networks and is independent of the selected routing approach.

The 6LBR is responsible for interconnecting the LoWPAN to the Internet and for disseminating IPv6 prefixes and header compression context information across the LoWPAN. The 6LBR also maintains a network cache of all IPv6 addresses and EUI-64 identifiers. In this way, 6LBR is able to make layer-two address resolution and to perform duplicate address detection (DAD).

Besides the Router Solicitation (RS), Router Advertisement (RA), Neighbor Solicitation (NS) and Neighbor Advertisement (NA) message types which were already defined for IPv6 networks, NDP for 6LoWPAN [6] defines two new ICMPv6 message types to implement DAD on multi-hop networks: Duplicate Address Request (DAR) and Duplicate Address Confirmation (DAC) and some new options on the previous message types.

**Figure 2.**
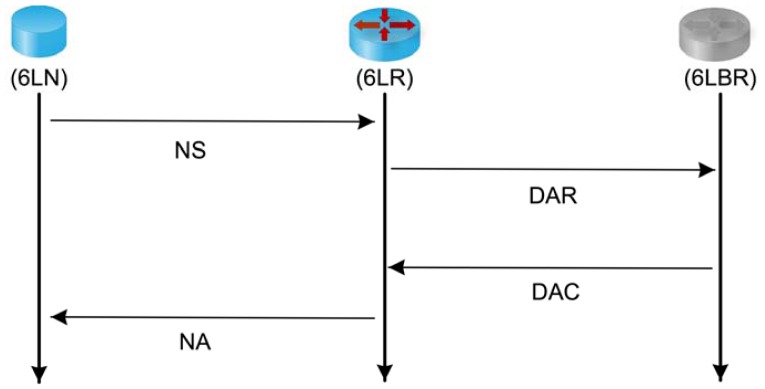
6LoWPAN neighbor discovery address registration.

When a 6LN interface is initialized, a link-local address is formed based on the EUI-64 [33]. Next, the 6LN multicasts a RS message indicating its source link-layer address. All 6LRs with direct connectivity reply with a unicast RA message indicating the available IPv6 prefix(es). Once an address has been configured, the following messages exchange is shown in Figure 2. First, a unicast NS message is sent to the selected 6LR to register its configured address (if 6LN receives RA messages from different 6LRs, it should attempt to register its address in more than one 6LN to increase the network resilience). Then, the 6LR sends a unicast DAR message to the 6LBR to check if the IPv6 address is already in use and the 6LBR replies to the 6LR with a DAC message indicating the status of the registration. The status indicates either a successful registration or a failure due to a duplicated address (or any other reason like, for example, the routers registration cache exhaustion). Finally, the 6LR sends a unicast NA message to the 6LN indicating the same status as received from the 6LBR in the DAC message.

The information contained on NA messages have an associated lifetime and the address registration process is repeated before the lifetime expires. In this way, NS messages are also used to perform unreachability detection and are mainly used by nodes to verify the default router reachability.

NDP is not secure when physical security on the link is not guaranteed. If a malicious node knows, by spoofing, the link-layer and the IPv6 addresses previously registered by a legitimate node, it might register the same IPv6 address either with its own link-layer address or with a fictitious address. This vulnerability can be exploited for different types of security attacks: redirection, denial-of-service and flooding denial-of-service. In redirection attacks, the malicious node receives the packets and reroutes them (either changed or unchanged) to their legitimate node. In denial-of-service attacks, the malicious node prevents communications between the legitimate node and either all other nodes or some particular destination node. In flooding denial-of-service attacks, the malicious node redirects the packets from other nodes to a victim node to create on it a flood of bogus traffic.

Meanwhile, the secure neighbor discovery protocol (SEND) [34] was proposed for traditional IPv6 networks to protect NDP against these attacks. The SEND protocol uses: (i) an authorization delegation discovery process to prove the routers' identity, (ii) an address ownership proof mechanism based on cryptographically generated addresses (CGA) and (iii) digital signatures for all NDP messages. SEND specification uses a cryptographically generated address method to bind a RSA public key to an IPv6 address and to digitally sign all NDP messages.

However, RSA is not suitable for low power and resource constrained nodes, because it is computationally intensive and leads to long message sizes [34]. To overcome this problem, the lightweight secure neighbor discovery for low-power and lossy networks work in progress specification (LSEND) [7] relies on elliptic curve cryptography (ECC) to generate the CGA and on elliptic curve digitally signature algorithm (ECDSA) to sign the NDP messages. In fact, elliptic curve cryptography can provide the same level and type of security guarantees as RSA using much shorter keys [34,35]. The computational overhead of both ECC and RSA raises with O(N^3^), where N is the bit length, while ECC and ECDSA lead to much smaller message sizes and lower computational load when compared with RSA [36]. All nodes generate a public and private key pair for each network interface in order to generate their own CGA addresses and to create the digital signatures [34], necessary to sign NDP messages. The digital signature is a hash code based on nodes private key, source and destination IPv6 addresses, header type protocol (8 bits), header checksum value (16 bits), the NDP message header (and its options) and a 128 bits constant, randomly generated and designated by message type tag. The ECC based algorithm used to generate CGA takes three parameters: the EUI-64 identifier, the public key of the interface and a three bit security parameter used to hamper brute force attacks.

Note that both SEND and LSEND protocols require no public key infrastructure. Therefore, any node, including potential attacker nodes, may generate and register valid CGAs, but an attacker node cannot use a CGA previously registered by legitimate nodes, preventing in this way the previously described attacks.

When a 6LN receives a RA message from a 6LR, it configures its own CGA address and launches the address registration process (as illustrated in Figure 2) by sending a NS message with both the configured address and the CGA options (*i.e*., the IPv6 source address of the NS message is set to its CGA address, the message carries the 6LN public key and it is signed with the 6LN private key). The 6LR receives the NS message and, based on its CGA options, runs two verification steps: (1) it verifies the source address using the claimed IPv6 source address and (2) it runs a cryptographic check of the signature included in the NS message. If both steps are successful, the 6LR proceeds with the address registration process as previously described. In this case, the 6LBR caches not only the 6LN IPv6 address but also its public key.

### 3.3. IPv6 Routing Protocol for Low Power and Lossy Networks

The IETF routing over low-power and lossy networks (RoLL) working group was chartered to design a routing protocol to be used in 6LoWPAN networks that addresses the requirements described in RFCs 5548 [37], 5673 [38], 5826 [39], and 5867 [40]. In 2010, RoLL introduced the IPv6 Routing Protocol for Low-power and lossy networks (RPL) [31].

Currently multipoint-to-point traffic pattern is dominant, because in most LoWPAN applications a few nodes are used to retrieve data from sensor nodes and the sensors rarely communicate between each other. To support this traffic pattern, RPL builds a destination oriented directed acyclic graph (DODAG) to route the data traffic. RPL defines a new ICMPv6 message with three possible types: DODAG information object (DIO) used to transport information that allows a node to discover an RPL instance, learn its configuration parameters and select DODAG parents; the DODAG information solicitation (DIS) to request a DODAG information object from a RPL node and the destination advertisement object (DAO) used to propagate information upwards along the DODAG.

During the DODAG construction and maintenance, nodes send DIO messages to their neighbors, carrying the objective function used to compute the rank value of each node. The rank defines the node relative position within a DODAG with respect to the root. The objective function specifies the metrics and constraints used to compute the routing path, the node rank position and the parent node set. RPL includes a flexible framework that incorporates dynamic routing metrics, such as expected number of transmissions (ETX). RPL also describes the constraints on how nodes select potential parents from their neighbors. For example, the node security support can be used as a constraint and, in this case, the nodes that do not support some security mechanism cannot be members of the DODAG. Nodes listen for DIO messages and use their information to join a new DODAG or to maintain an existing DODAG. Based on the DIO information, nodes choose parents that minimize path cost to the DODAG root. In the steady state, each node has a set of parents where the one with least rank value is selected as the preferred parent and the others are used as backup nodes.

DIO messages only enable upward routes computation and nodes have no knowledge about their children. In order to support routing to other destinations within the DODAG, RPL uses DAO messages to inform parents of their presence and reachability to descendants. The root node gathers the DAO messages from all other nodes and uses them to build downward routes to all destinations. RPL also defines local and global repair methods for re-computing routes when some inconsistency is detected.

Security is an important design consideration for LoWPAN networks because several attacks against the routing protocols were already identified. In order to guarantee the integrity of routing messages, RPL defines an optional cryptographic operation mode, in which advanced encryption mechanisms are used for message authentication. AES for message authentication and RSA signatures for checking the integrity of routing messages are already considered. Using ECC signatures to substitute RSA is possible and desirable because it provides the same security guarantees with much smaller keys, although the ECC inclusion is a work in progress [41].

### 3.4. Node Remote Reprogramming Mechanisms

In the general case, wireless node reprogramming, also named over-the-air reprogramming, is a useful tool for remote uploading new codes or for changing the functionalities of existing codes [42]. Remote reprogramming is especially useful on large networks installed on harsh or difficult access environments. In our case, the proposed network access control framework relies on remote reprogramming as a means to enforce node security, *i.e.*, to guarantee that legitimate nodes are using security free codes before being connected to the network.

Remote software installation is more challenging on LoWPAN networks mainly because of the resource constraints of the nodes, the fact that a single network might have thousands of nodes and the fact of communications being over multihop links. Moreover, a program image is relatively large to be transmitted over low-power wireless links where link losses and collisions often occur. Consequently, efficient mechanisms must be used to upload the software, minimizing the nodes energy consumption. Significant research has been done in order to address the resource constraints nature of LoWPAN networks. The first proposed reprogramming mechanisms have only assumed single-hop networks. In this case, only nodes with direct links with the border router (usually selected to store the new software because it has more resources than the other nodes) can be reprogramed. Recently, more mechanisms were proposed to address multi-hop reprogramming [43]. In fact, multi-hop reprogramming can be more efficient, both in terms of time and energy, when compared with single-hop reprogramming. First, shorter links can be used, which reduces the retransmissions due to collisions. Second, the multi-hop protocols divide the entire code image into pages and when a node completes downloading a single page, it can send it to other nodes in the network while downloading the next page (this process is referred to as spatial multiplexing).

**Figure 3.**
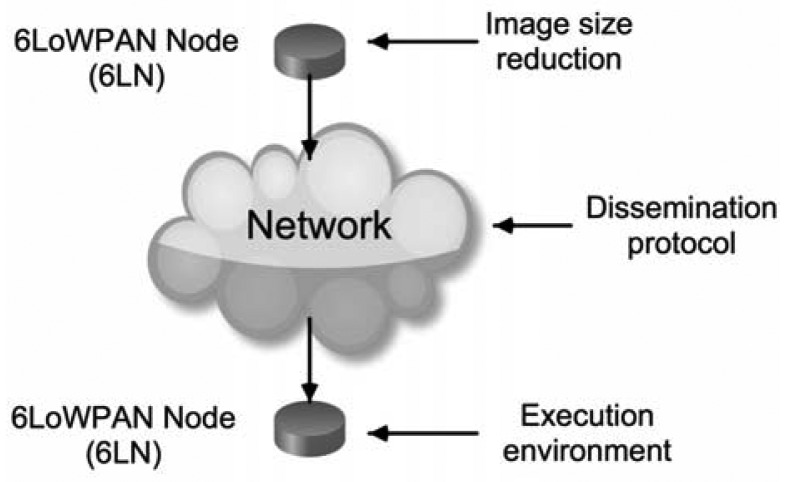
Node remote reprogramming mechanisms.

Remote reprogramming research is organized under the following three categories [44] (Figure 3): the sensor node execution environment, the protocols for update dissemination, and the image size reduction. Power efficiency, performance, security and reliability are common issues addressed by the solutions proposed for each category.

#### 3.4.1. Sensor Node Execution Environment

The execution environment is the responsible to run programs on top of the available hardware. The first systems had dedicated execution environments to make the best use of the available hardware heterogeneity. The presence of a memory management unit (MMU) can have a significant impact on the software update process. The MMU is the hardware component that manages virtual memory systems and, in most situations, it is included on the CPU chip. When the MMU is available, much of the code position is independent, because virtual memory addressing is used, adding safety boundaries to programs. Moreover, individual modules are executed as separate processes. Since processes run in a virtual address space, they can be easily upgraded at runtime without using references to absolute memory addresses. However, many of microcontrollers used on sensor nodes are not equipped with MMU. Without the MMU, a node operates in a single address space and, in such case, the node is more vulnerable to incorrect or malicious memory references. Execution environments without MMU can be classified in monolithic environments, modular environments and virtual machines.

In monolithic environments, the entire executable program is statically optimized during the compilation. In this case, a single system image containing both all applications and the system kernel is produced making efficient use of CPU and memory. The TinyOS operating system is an example of a monolithic execution environment. In such execution environments, a new complete image must be uploaded into the node, resulting in large update patches.

In modular execution environments, individual modules can be independently loaded on demand. In this type of environment, the system is divided into two parts: the kernel, usually static, and the loadable component images, usually dynamic. The kernel provides services to the modules, such as memory management, I/O, and communications. The modules access the services provided by the kernel at predefined addresses or through a system jump table. The updates are smaller than in monolithic counterpart because the modules are separated from the kernel part. Moreover, the kernel part requires less frequent updates than in monolithic environment because it only provides the interfaces to the module applications. Contiki and Bertha operating systems use modular execution environments.

Virtual machine (VM) environments are used to virtualize underlying hardware, providing high-level operations to applications through an instruction interpreter. VM typically executes a program in a sandbox where direct access to hardware is not allowed. In sensor networks, a VM environment also allows the implementation, by software, of some features that the hardware might not provide, such as the memory management unit. Unfortunately, VM introduces overhead in both execution time and memory resources. First, the runtime interpretation causes programs to run slower by at least an order of magnitude when compared with native execution. Second, the VM itself requires a certain fixed amount of memory to operate, and interpreted programs are generally more memory demanding. Several VM environments for TinyOS and Contiki operating systems were proposed, such as Maté and Agilla [45].

#### 3.4.2. Protocols for Update Dissemination

The proposed protocols for software update dissemination are mainly based on data dissemination protocols, such as RMTS [46] and direct diffusion [47]. Software dissemination protocols operate in three steps: (i) advertisement of available software, (ii) source selection and (iii) reliable download to the destination.

Deluge is generally accepted as the state of the art for code dissemination in wireless sensor networks, and has been included in the latest TinyOS distributions [48]. Deluge is a reliable data dissemination protocol for disseminating data objects from one or more source nodes to many other nodes over a multihop wireless network. Data objects are represented as a set of fixed size pages to enable incremental updates and to allow spatial multiplexing.

Code dissemination protocols can be used to compromise LoWPAN networks. For example, an attacker may attempt to modify or replace the authentic code image introducing malicious code into the network nodes. Code dissemination protocols can also be used to perform denial-of-service attacks, where the malicious node injects bogus code and force network nodes to verify and forward them leading to exhausting their battery power. Several recent protocols, based on Deluge, were proposed to address secure code dissemination. For example, Sluice integrates cryptographic digital signatures and hash functions to provide authentication for code dissemination. Like Deluge, Sluice also splits code images into fixed size pages. The hash code of each page is included in the previous page. The hash code of the first page is signed and included in the packet signature. This approach solves the authenticity attacks, but it does not address denial-of-service attacks. Since a node can only perform authentication when a complete page is received, many packets must be processed before the authenticity can be verified. Seluge [49,50] inherits the efficiency and robustness properties from Deluge providing authentication mechanisms for code dissemination and protection for DoS attacks against signature packets, code dissemination packets and maintenance packets. Seluge uses public key mechanisms based on elliptic curve cryptography.

#### 3.4.3. Size Reduction Mechanisms

While the dissemination protocol aims to minimize the overhead related to delivering updates, reducing the size of transmitted software is used to decrease the size of the updates. Three main techniques are used to achieve this objective: compression, differential patching and high-level instructions. Several reducing size mechanisms were proposed, such as: Reijers [51], Rsync [52] and Remote Incremental Linking [53]. Reijers reduces the image size generation differential script. The script is downloaded as a series of packets where each packet contains the new instruction code and its address. The script is applied against the currently running program stored in the external memory, usually an EEPROM, gradually building a new image. When this process is completed, the boot loader loads the new image into the running memory. The scripting language is cpu-specific, which is the Reijers algorithm major flaw. Rsync also provides incremental software updates but its algorithm is CPU independent. The Rsync script can either specify unmodified block to be copied for a new address or can contain modified code. The updater node asks the node being updated for the checksum of its current blocks and, according to the response, only sends changed code. Remote Incremental Linking was designed for mica2 mote hardware platform and supports dynamic and static updates. An image program is composed by several functions and at the end of the functions some space is reserved to allow future expansion. New versions of the functions can be written at the same start address memory, as long as they fit, without the need to update references to the functions elsewhere in the program. Otherwise, either adjacent functions or the new functions are moved, which minimizes page writes.

## 4. Network Access Control Security Framework

This paper proposes a network access control security framework, for 6LoWPAN networks, that controls the access of nodes to the network, based on administrative authorization, and enforces security compliance to the authorized nodes. The proposed framework can operate in two different modes: listening and active. In the listening mode, no security assessment and enforcement is performed. This mode is useful for network visibility, because it can be used to gather information about the connected nodes. The active mode is more secure than the listening mode because only security compliant nodes can be connected to the network.

### 4.1. Nodes Requirements

In order to provide a secure and manageable solution, a few assumptions related to nodes and border routers must be defined, according to the selected operating mode. To operate in listening mode, all nodes must support 6LoWPAN and LSEND protocol. To operate in active mode, in addition to the previous requirements, the nodes must also support a secure reprogramming mechanism and the RPL routing protocol with message authentication. The secure reprogramming mechanism can be implemented using Seluge combined with Maté execution environment and Rsync size reduction mechanism. The border router must support both operating modes simultaneously and, therefore, their requirements are the same as the nodes when supporting the active mode. We assume that border routers are hard to be compromised.

### 4.2. Node Identification, Compliance and Data Security

As sensor networks are mostly deployed in human-unattended environments, usually for critical sensing measurements tasks, the authentication of the data source as well as the data itself is of critical concern. In fact, authentication guarantee can provide both sensor and router identification ability, in order to protect the integrity and freshness of critical data, and forbid and/or identify several security attacks. Traditionally, there are two schemes to provide authentication: digital signatures based on public-keys and message authentication code based on symmetric-keys. In the current proposal, the same public and private key pairwise, generated by the LSEND protocol to build the cryptographically generated addresses (CGA) addresses, is used to authenticate the network nodes. As aforementioned, the CGA are IPv6 addresses where the least 64 address bits are generated by computing a cryptographic one-way hash function from a public key and other auxiliary parameters. The address owner uses the corresponding private key to declare the address ownership and to sign the messages sent without a certification authority or other security infrastructure. The binding between the address and the public key can be confirmed by re-computing the hash value and by comparing the hash with the interface identifier. As a consequence, messages sent from a CGA IPv6 address can be protected by attaching the public key and auxiliary parameters and by signing the message with the corresponding private key. Every node can generate valid private and public key pairwise and the correspondent CGA address and, once registered in the border router, no other node can use the same address. In our proposal, after the LSEND registration procedure, the administrator approves manually each new node based on its CGA address. Once authenticated, each node is able to verify if the sender node is the right owner of the CGA generated source address. One of the key advantages of this approach is that, in addition to ensuring the identity of the sender, the same public and private pairwise can be also used to guarantee the authenticity, confidentiality and data freshness to all protocols or mechanisms in use.

Traditionally, two different approaches are used to perform node security compliance. In the first approach, a piece of software named agent is used to perform the nodes assessment. The agent returns to the security enforcement point (in our case, the border router) the result of the assessment. In the second approach, the assessment is performed based on the traffic generated by the node under assessment. The security enforcement point sends requests to the node under assessment and the evaluation is made based on the responses. Usually, the first approach returns more accurate results than the second approach. Moreover, the second approach is more verbose than the first one, because multiple messages are exchanged. The current proposal adopts the agent based assessment approach. The agent can be previously installed on the nodes and the assessment is based on cryptographic hash of the installed software. If the agent is not installed, we assume that the node is not compliant and a new image, which includes the assessment agent, will be uploaded by the reprogramming mechanism on the fly. The hardware configuration can also be evaluated during the assessment, although this feature is not considered in our solution. Note that any solution to perform nodes security assessment is very dependent on the operating system and the hardware used in the network devices. So, different agent versions might be required if different operating systems or hardware platforms are to be used. The border router can use operating system fingerprinting in order to determine which agent version has to be used.

### 4.3. Access Control Algorithm Description

**Figure 4.**
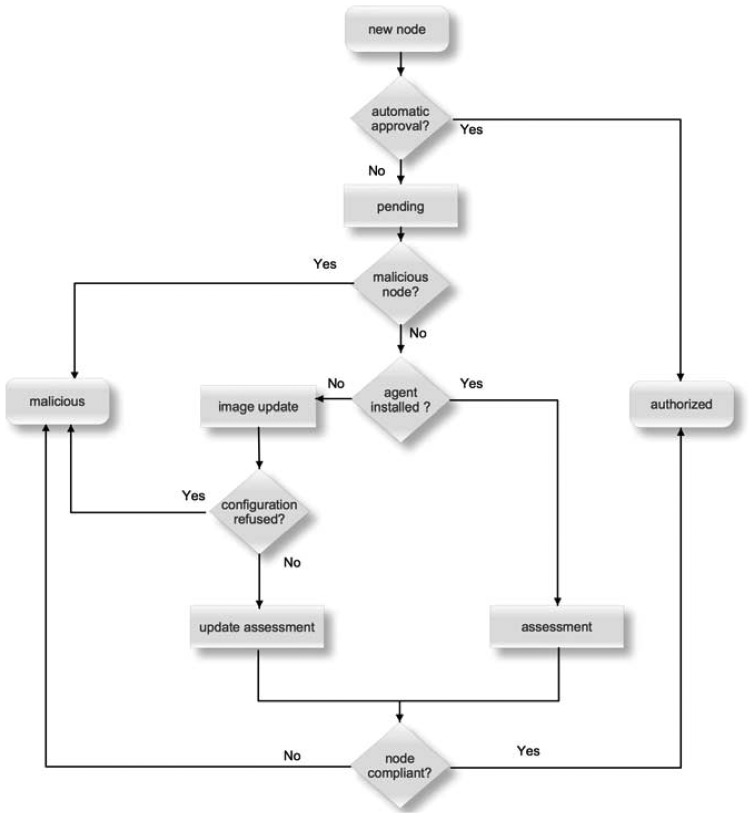
Access control decision process.

The access control algorithm of our proposal assumes that each node is always in one of seven states: new node, pending, assessment, image update, update assessment, authorized and malicious. Figure 4 illustrates the decision process from the initial ‘new node’ state until one of the final states is reached, which is either ‘authorized’ or ‘malicious’.

The 6LoWPAN nodes can use link layer detection mechanisms and/or router advertisement messages to detect new networks. If a new network is detected, the 6LoPWAN nodes must use LSEND protocol to perform the address registration process, as described in section 3.2. The address registration process is used to detect new nodes presence. In the listening mode, the automatic approval is in use and, therefore, the new node moves to the authorized state and its address is added to the border router as a registered entry [6]. This is the less secure operation mode and is used by the administrator to determine which nodes are connected.

In the active mode, the automatic approval is not set and, therefore, the new node moves to the pending state and its address is stored in the border router neighbor cache as a tentative entry [6]. Before the authorized state is reached, only messages related to the assessment, image deployment and authorization are allowed. In the pending state, the administrator can classify the new node as malicious if: (i) additional nodes are not expected, (ii) the administrator does not rely on the received information or (iii) the new node was considered malicious in the past. If the administrator does not consider the new node as malicious, the next step is to verify if the assessment agent is installed. If yes, the node moves to the assessment state and its security compliance is evaluated. Then, the node transits to the authorized state if the evaluation is successful or to the malicious state, otherwise. If the assessment agent is not installed, the node moves to the image update state, where the border router tries to install on the node a new image using Deluge (the new image includes the operating system, the assessment agent and all required modules). If the configuration of the new image is refused by the node, if moves to the malicious state. Otherwise, the node moves to the update assessment state and its security compliance is evaluated. Then, the node transits to the authorized state if the evaluation is successful or to the malicious state, otherwise.

When a node is moved to the malicious state, its address is marked as garbage-collectible in the border router neighbor cache, the address is propagated by the RPL messages to all authorized nodes as a restriction, and the address is pruned from all nodes RPL candidate neighbor list and cannot belong to the DODAG instance in use. So, in the malicious state, no messages from these nodes are processed or forwarded by any other LoWPAN node.

## 5. Discussion

The security research applied to LoWPAN networks is considered by the industry and by the research community a very hot topic. Several security mechanisms were proposed, some of them to address some particular scenarios, application and protocols. Besides protecting the network from some well-known threats, it is important that security mechanisms can withstand to attacks that have not been identified before. Therefore, the challenge is on how can the attackers explore vulnerabilities that have not been yet identified. The control of which nodes can take part of the network and their security compliance can help to reduce several vulnerabilities, making the network more manageable, while increasing its reliability and extending its lifetime. The proposed security framework proposal can be used to achieve these objectives, in particular to make the network more resilient to internal attacks. First, network assessment control restricts the network access only to authorized nodes. Second, node security compliance is enforced before node can access to the network. Finally, beyond to security compliance assessment, the software image can also be checked in order to guarantee that node is able to realize the expected functions. Therefore, only authorized nodes that fulfill security and functions requirements are accepted. This is particularly relevant if multi-hop networks are used. Node authorization depends on:

- Administrator authorization: a manual authorization was considered because it is very hard to define rules that can be applied to all network security requirements. For example, in a monitoring network installed in a nuclear power plant, if a new node tries to access the network, it will be most probably a malicious node since the network infrastructure remains unchanged for long time periods. Therefore, the administrator can approve the new nodes based on: hardware type, layer-two address and location. This approval method also protects the framework against DoS attacks, because only approved nodes will be evaluated. All nodes are identified by a cryptographic generated address, according to LSEND protocol.
- Security check compliance: several conditions can be considered as inputs to the agent used to assess the security compliance such as, for example, the installed software image and the security protocols in use. Note that multiple agents might be required if different operating systems or hardware platforms are used in the same network. The decision on which agent should be used on each device node is a challenge.
- Hardware and software image compliance: providing plug-and-play mechanism is not enough to guarantee that node is able to realize the desired functions. For example, a sensor node is unable to monitor the temperature if the module used to retrieve the temperature is missing. The same occurs with the hardware. Software image compliance also helps to protect against malicious code injection.

The proposed framework also improves the network manageability, since remote software installation is supported. The implementation success of the proposed security framework depends on the ability to integrate the above described mechanisms and protocols in order to take advantage of synergies between them. In fact, the following modifications are required in order to maximize the integration benefits.

LSEND requires the addition of a secure mechanism to inform nodes that a neighbor must be removed from its neighbor cache in order to avoid communications between authorized nodes and malicious nodes. It is also necessary to define a data structure to share the ECC key pairwise generated by the LSEND protocol with the RPL routing protocol and remote image installation mechanism. Note that the ECC key pairwise can also be used to protect the application layer data exchange, in order to guarantee data authentication and authenticity. In fact, the reutilization of the same ECC private and public key pairwise between several protocols and mechanisms simplifies the operations related to the key management [25]. The key pairwise reutilization also extends the network lifetime because fewer messages are used when compared with other solutions that use one key for each protocol or mechanism. In fact, the transmission energy consumption rate, in wireless sensor networks can be over three orders of magnitude greater than the energy consumption rates for computing [54].

In the RPL protocol, the ECC support must be defined in order to protect the routing messages exchange. Also, an efficient mechanism must be defined in order to propagate efficiently addresses as a constraint in order to avoid the malicious nodes to participate in the routing tree.

Concerning node remote reprogramming mechanisms, several mechanisms must be combined to enable node remote reprogramming. These mechanisms are very dependent on the operating system and the hardware in use. As a consequence, it is foreseen that early implementations of the proposed framework in real deployment scenarios will support node remote reprogramming mechanism only for a few hardware platforms and operating systems. Currently, a laboratory testbed is being implemented where the above-mentioned protocol modifications are being conducted and integrated aiming to validate the proposed security framework.

## 6. Conclusions and Future Work

Improving the security is critical for the success of LoWPAN networks, because these types of networks are particularly vulnerable, they are used in critical services and the data collected is often sensitive. Several security solutions were proposed, most of them designed to address known attacks and implemented on particular layers. Several security attacks can be avoided if a network access control mechanism is used to restrict the network access only to authorized nodes which are compliant with the defined security requirements. This paper, we have proposed a network access control framework that can be used to accomplish these objectives. The proposed solution enables the node identification based on cryptographically generated addresses, node security compliance evaluation and node remediation with secure remote software installation. This solution is mainly based on the following open protocols: LSEND, used to secure neighbor discovery and node secure identification, RPL with ECC support, to protect the routing messages and Seluge, to enable secure remote software installation. In the current proposal, synergies between the protocols were taken in consideration. Work is still required to improve and integrate these protocols in order to create a solution. Moreover, the current proposal only enables one border router. As a consequence, when the border router becomes unreachable, all nodes must be approved and evaluated. Further research is required to circumvent this limitation since a secure mechanism is required to synchronize the ECC keys and nodes authorization state between multiple border routers. Besides the current lab implementation to validate our current proposal, this research is another direction of our future work.

## List of Acronyms

6LN: 6LoWPAN Node
6LR: 6LoWPAN Router
6LBR: 6LoWPAN Border Router
AES: Advanced encryption standard
CGA: Cryptographically generated addresses
DAD: Duplicate Address Detection
DAG: Directed Acyclic Graph
DAR: Duplicate Address Request
DODAG: Destination Oriented DAG
ECC: Elliptic curve cryptography
LSEND: Lightweight Secure Neighbor Discovery for Low-power and Lossy
MAC: Medium Access Control sub-layer protocol
MTU: Maximum Transmission Unit
NA: Neighbor advertisement
NDP: Neighbor discovery protocol
NS: Neighbor solicitation
PHY: Physical layer protocol
RA: Router advertisement
RPL: IPv6 Routing Protocol for Low-power and lossy networks
RS: Router solicitation
SEND: Secure neighbor discovery protocol
